# Modern Treatment of Headache

**Published:** 1891-03

**Authors:** 


Modern Treatment of Headaches. By Allan McLane Hamilton, M. D. The Physician's Leisure Library. No. 6, second edition. Detroit: George S. Davis. 1890. Price, cloth, 50c.; paper, 25c. Pp. 122.

If a physician peruses this little work during his leisure moments, he will find it not only a pleasure but an important aid in the treatment of this obstinate ailment. It is overflowing with valuable suggestions and practical formulæ drawn from the author's own experience, that consequently carry with them much weight and conviction. The fact that this work has undergone a second edition shows it to be fully appreciated by the profession at large.

